# Self-powered, ultrasensitive, room temperature humidity sensors using SnS_2_ nanofilms

**DOI:** 10.1038/s41598-020-71615-5

**Published:** 2020-09-03

**Authors:** A. Rambabu, Deependra Kumar Singh, Rohit Pant, K. K. Nanda, S. B. Krupanidhi

**Affiliations:** 1 Department of Basic Sciences and Humanities, GMR Institute of Technology, Rajam, Andhra Pradesh 532127 India; 2 Quantum Structures and Device Laboratory, Materials Research Centre, Indian Institute of Science, Bangalore, 560012 India

**Keywords:** Materials science, Materials for devices, Nanoscale materials

## Abstract

Humidity monitoring has become extremely vital in various technological fields such as environment control, biomedical engineering, and so on. Therefore, a substantial interest lies in the development of fast and highly sensitive devices with high figures of merit. Self-powered and ultrasensitive humidity sensors based on SnS_2_ nanofilms of different film thicknesses have been demonstrated in this work. The sensing behavior has been investigated in the relative humidity (RH) range of 2–99%. The observed results reveal a remarkable response and ultrafast detection even with zero applied bias (self-powered mode), with response and recovery times of ~ 10 and ~ 0.7 s, respectively. The self-powered behavior has been attributed to the inhomogeneities and the asymmetry in the contact electrodes. The highest sensitivity of ~ 5.64 × 10^6^% can be achieved at an applied bias of 5 V. This approach of fabricating such highly responsive, self-powered and ultrafast sensors with simple device architectures will be useful for designing futuristic sensing devices.

## Introduction

Humidity monitoring and control plays an essential role in maintaining the safety and quality of day-to-day life as it has applications in many fields, including environmental control, industrial production, medical and chemical monitoring, and so on^1,2^. In recent years, many efforts have been made to develop humidity sensors based on various transduction principles such as capacitive, resistive, acoustic and optical detection^3^. Nanoscale materials have been investigated for further improvement in sensing performance in terms of higher sensitivities, and faster response and recovery times. However, all these sensors require a power supply and therefore, in a device which involves a number of such sensors, power supply becomes a huge concern which can lead to incredible cost and complexity. In the current energy scenario, considerable efforts are being made in the fields of energy storage and energy generating devices^4–11^. Hence, low cost and self-powered nano-devices are desperately in demand.

It is well established that metal dichalcogenides (MDCs) have been widely investigated for various applications such as photocatalysis, optoelectronics, solar cells, sensors, etc. due to their extraordinary properties and performance in nano-electromechanical devices^1,12–16^. Among these MDCs, earth abundant layered SnS_2_ is being extensively investigated due to its additional advantages such as non-toxicity, high stability, and most importantly, low fabrication costs^17^. Therefore, in recent times, SnS_2_ based devices have become potential and important candidates for gas and humidity sensing. Bharatula et al. have synthesized nanofilms of SnS_2_, which can effectively detect humidity with response and recovery times of ~ 85 and ~ 6 s, respectively^18^. Qin et al. have demonstrated SnS_2_ nanosheets prepared by chemical exfoliation, which exhibit rapid response and high sensitivity to NH_3_ at room temperature^19^. Zhang et al. have demonstrated a self-assembled SnS_2_ nanoflower/Zn_2_SnO_4_ hollow sphere nanohybrid device, exhibiting excellent humidity sensing properties^20^. Recently, Yan et al. fabricated NO_2_ gas sensor based on CVD grown SnS_2_ with ultra-high sensitivity by utilizing inherent defects under photoillumination^21^. Although, there are many reports on SnS_2_ based devices for humidity and gas sensing, the quest is always on for fabrication of devices with properties such as large area with simple designs, self-powered, ultrasensitive and room temperature humidity sensing.

There are several techniques to obtain low-dimensional SnS_2_ thin films. Exfoliation, chemical vapour deposition (CVD), hydrothermal and solvothermal synthesis, and sputtering have been extensively used for fabricating nanofilm based devices^20–24^. However, exfoliation of nanoflakes and growth of thin films by CVD result in thin films whose sizes are in the range of few μm, thus hindering the large-scale growth optimum for industrial scale applications. Additionally, absence of ultrahigh vacuum during fabrication can often lead to unclean and contaminated interfaces. Therefore, magnetron sputtering has emerged as a perfect tool for deposition of large-scale thin films, involving ultra-high vacuum, which helps in controlling the thin film properties during deposition.

Herein, we demonstrate self-powered humidity sensing based on SnS_2_ nanofilms and the effect of thickness modulation on the sensing behavior. The concept of self-powered detection lies with the presence of an internal electric field, which is strong enough to separate the charge carriers^11^. This phenomenon has been utilized in the designing of sensing devices. The self-powered detection can be realized by several approaches such as fabricating hybrids with piezoelectric materials, or depositing asymmetrical contact electrodes of different materials^25,26^. The most convenient way for realizing self-powered response is by introducing asymmetry in the contact electrodes of same material^27^, and this property has been exploited in the present study. The nanofilms have been prepared by a two-step growth process: deposition of Sn thin films on soda lime glass (SLG) substrates by dc magnetron sputtering followed by their sulfurization. Humidity sensing have been carried out at room temperature and the devices exhibit excellent detection even at zero bias, with ultrafast response. The sensitivity is found to be optimum for a film thickness of 262 nm. A possible mechanism based on charge hopping has been proposed to have an understanding about the detection phenomenon.

## Results and discussion

**Figure 1:**
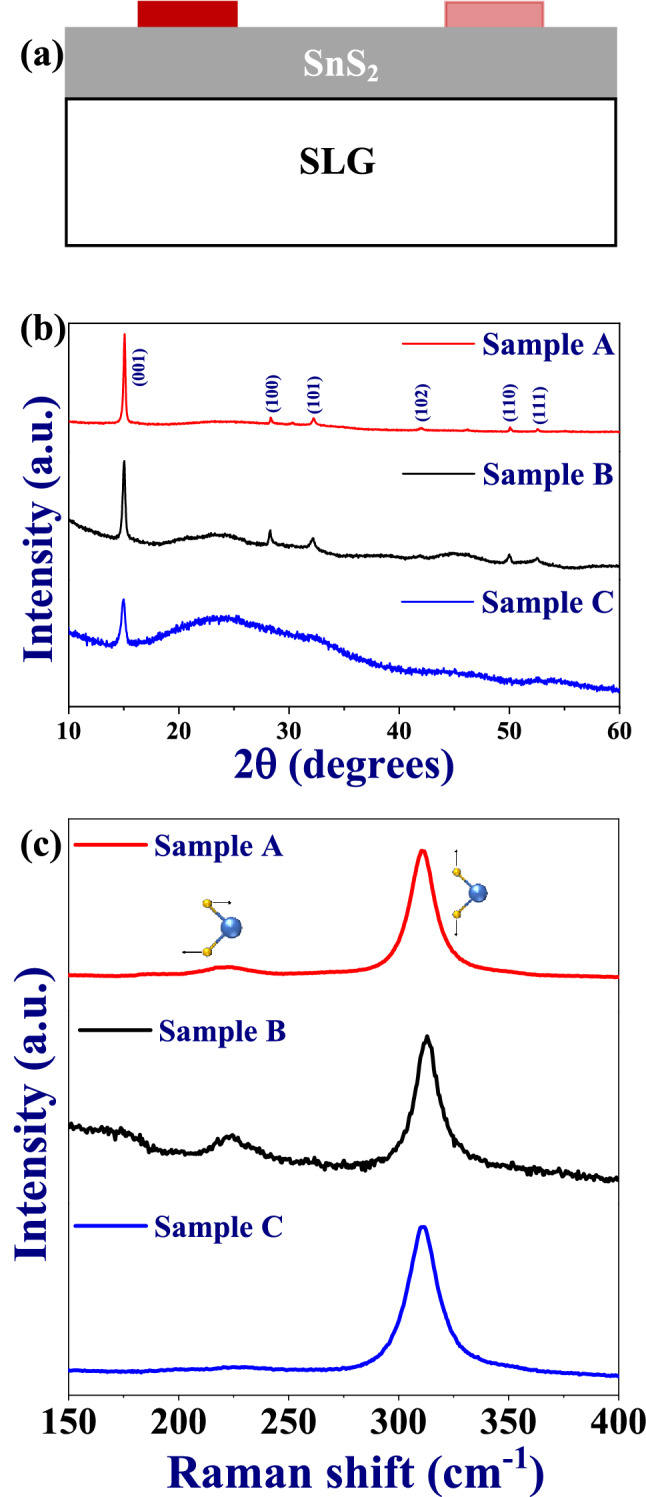
(**a**) Schematic of the fabricated device with two inhomogeneous/asymmetric electrodes, (**b**) XRD patterns, and (**c**) Raman spectra for sample A, sample B and sample C.

Figure 1a shows a typical schematic of the SnS_2_ based humidity sensor. For investigation of the crystalline nature and phase purity of the thin films, X-ray diffraction (XRD) patterns were recorded and the patterns are shown in Fig. 1b. The peaks have been indexed in accordance with the JCPDS (22-0951), which confirmed the formation of hexagonal pure phase of SnS_2_ with space group P3m1^28^. The peak corresponding to (001) is found to be the most intense peak, thereby indicating z-direction oriented growth. As the thickness of the film increases, the intensity of the (001) peak becomes stronger. Moreover, the hump observed ~ 25° corresponds to the amorphous nature of SLG and decreases with increasing film thickness. The crystalline nature of the films is confirmed by Raman spectroscopy and the spectra have been presented in Fig. 1c. The two signature vibrational modes A_1g_ (~ 312 cm^−1^) and E_g_ (~ 224 cm^−1^) can be clearly seen for all the three samples, which correspond to the vertical out of plane vibrations and the non-degenerate in-plane vibrations, respectively^18^. Thus, the formation of crystalline SnS_2_ thin films on SLG substrate is confirmed.

**Figure 2:**
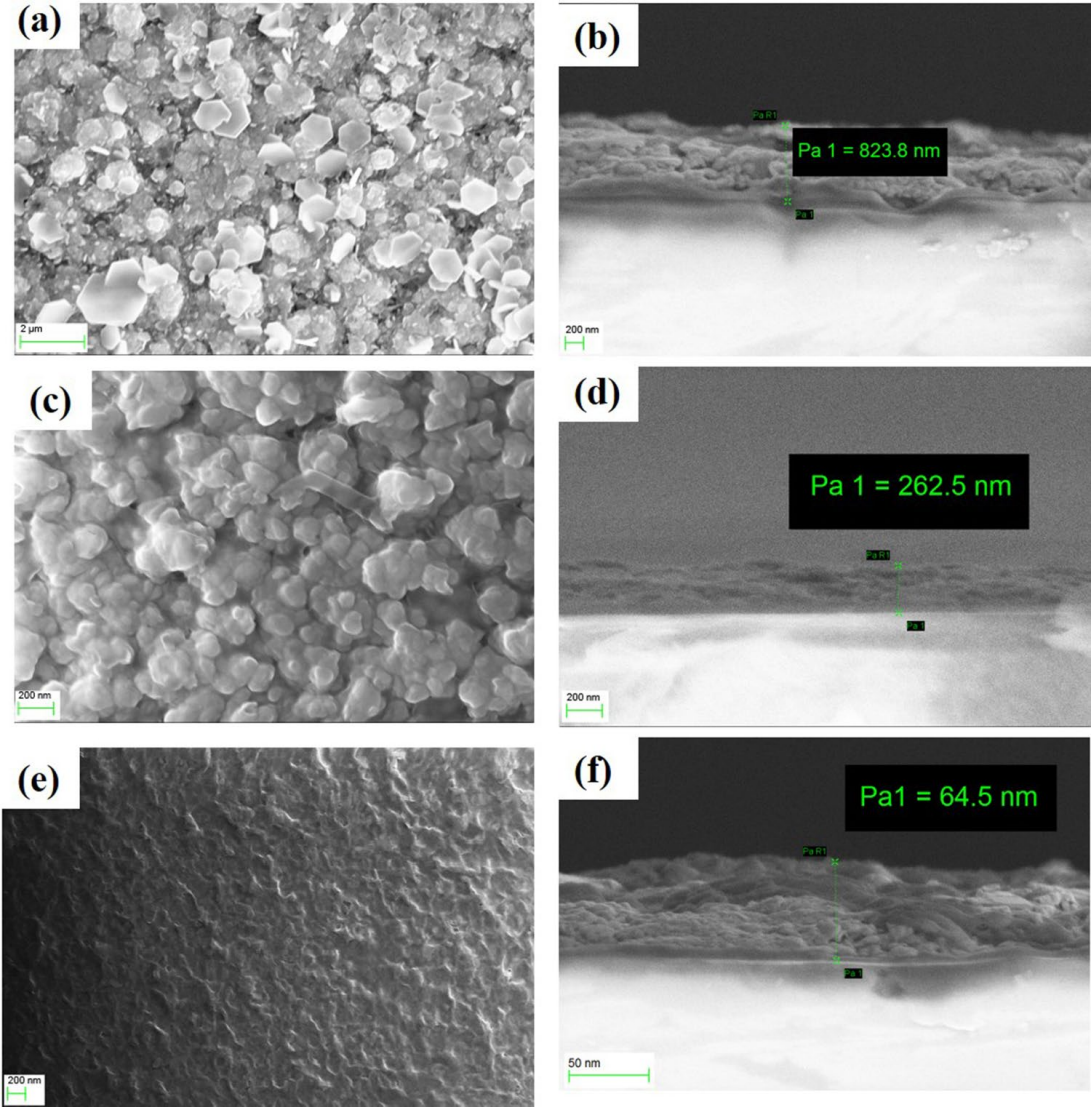
Top surface and corresponding cross-sectional SEM images of (**a**,**b**) sample A, (**c**,**d**) sample B, and (**e**,**f**) sample C.

The surface morphology and the cross-sectional scanning electron microscopy (SEM) images of the SnS_2_ thin films are shown in Fig. 2. The top surface SEM micrographs reveal a granular structure for all the samples (Fig. 2a,c,e). From the thickness measurements, the thicknesses of the samples were found to be ~ 823, ~ 262 and ~ 64 nm for sample A, sample B and sample C, respectively (Fig. 2b,d,f). The EDS spectra for all the thin films have been presented in Figure S1 of the Supplementary Information that confirmed its parent elements with uniform and homogenous distribution throughout the sample.

**Figure 3:**
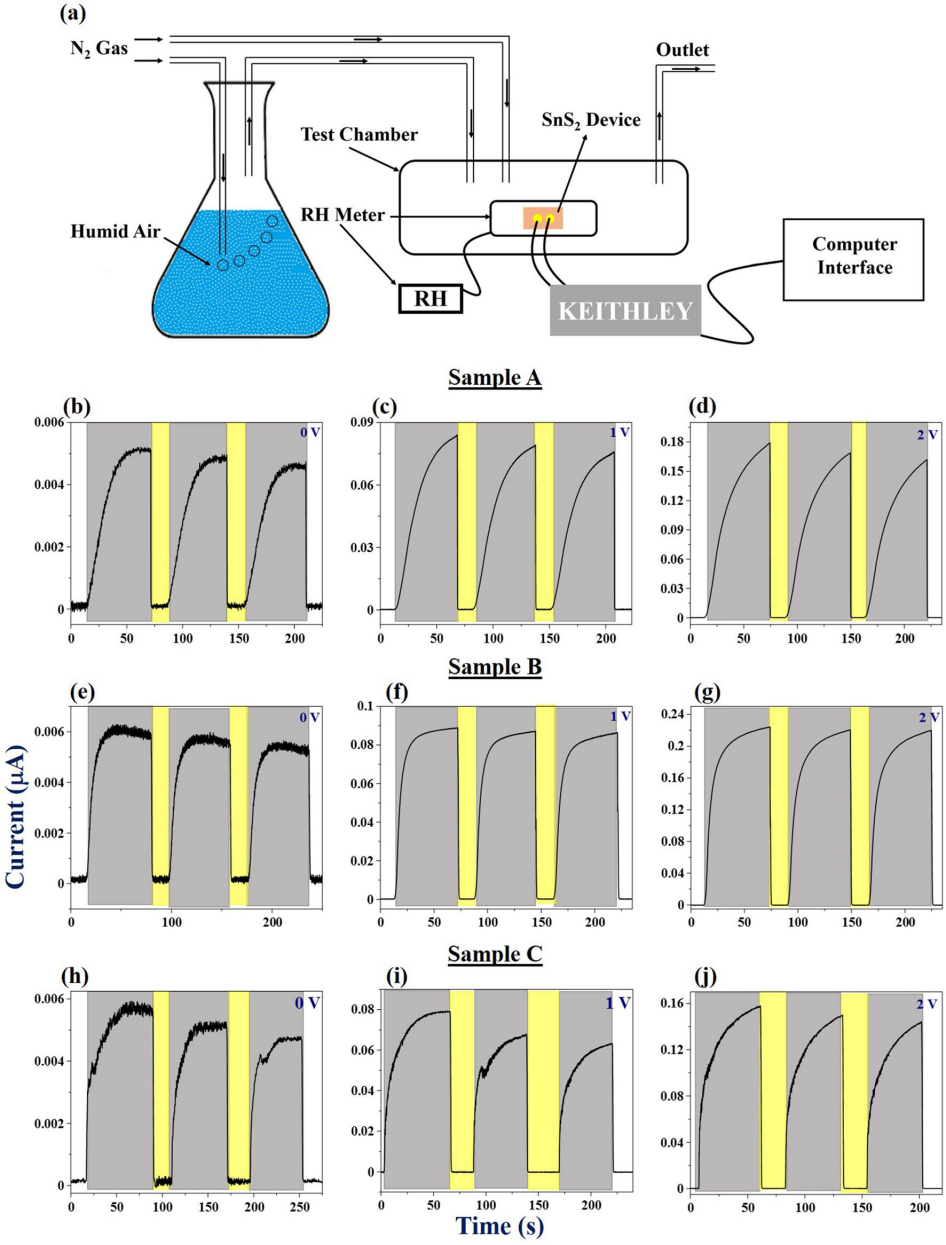
(**a**) Schematic of humidity sensing setup. Temporal response curves for (**b**–**d**) sample A, (**e**–**g**) sample B and (**h**–**j**) sample C at different biasing voltages (as indicated in the plots).

The humidity sensing behavior (at room temperature) of the devices has been investigated in the range of 2% (dry N_2_ environment)–99% (highly humid atmosphere) with the help of a lab-built setup (Fig. 3a). The results have been depicted in Fig. 3b–j. The temporal response (*I–t*) curves of the devices under the influence of different biasing conditions (0–2 V) were recorded to examine the response/recovery times and the cyclic stability of the devices. It was observed that the current increases as the humidity level is increased and gradually saturates. However, there was a rapid drop in the current as the humidity is lowered by flushing the chamber with dry nitrogen. The sensors were tested with different biases and similar kind of behavior was observed.

**Figure 4:**
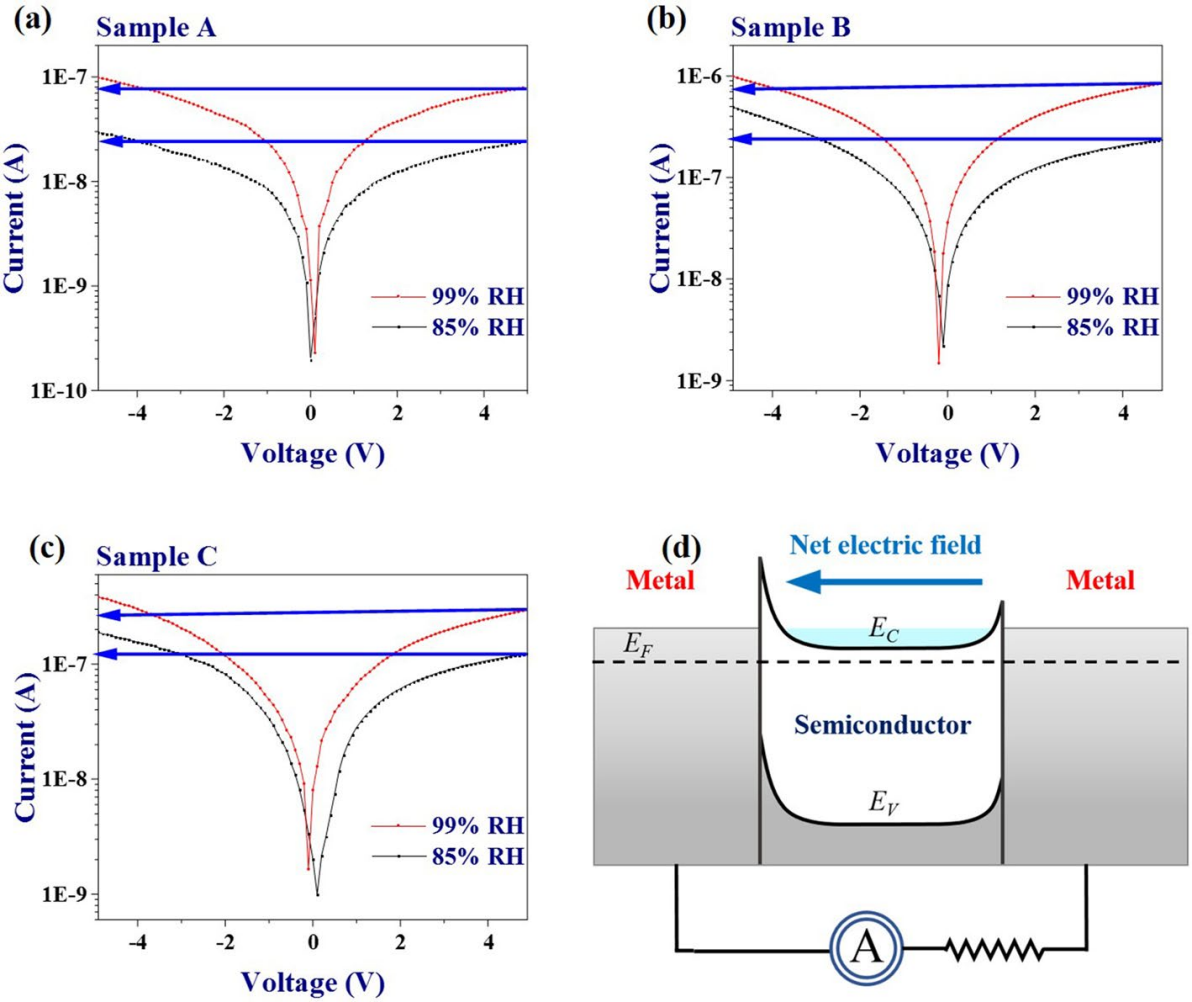
(**a**–**c**) *I − V* plots of the three devices (as indicated in the plots) taken from − 5 to 5 V for the confirmation of the inhomogeneous and asymmetric nature of contact electrodes at two different %RH levels (99% and 85% RH). (**d**) Net internal electric field due to asymmetry in electrodes, which results in self-powered detection.

It is worthy to note that the sensors respond even at zero bias condition, thus depicting self-powered nature. The possible reason for this self-powered response could be the asymmetry and inhomogeneity in the contact electrodes as mentioned in the introduction. The fabricated Cr/Au electrodes are supposed to be homogenous and symmetrical in nature. It appears from SEM micrographs that all the films are rough. Therefore, the deposited electrodes are expected to have unintentional inhomogeneities in them. For further confirmation, we have shown the current–voltage (*I–V*) graphs of the three devices at two different humidity levels (85% and 99% RH). The *I–V* characteristics of all the devices have been shown with respect to the same values of the negative and positive voltage (Fig. 4a–c). It is observed that all the devices show significant asymmetry in terms of current at different humidity levels. The current is different for the same positive and negative biases confirming the overall asymmetry in the device (marked by arrows). These inhomogeneities in contacts and at the interface would result in different barrier heights at the two electrodes (Fig. 4d). Different barrier heights signify that the built-in electric fields at the metal/semiconductor junction will be different, thus resulting in a net internal electric field that is responsible for self-powered sensing^29,30^.

The response (t_r_) and recovery (t_f_) times have been evaluated by estimating the difference in time for the signal to rise or fall from 10 to 90% or 90% to 10% of the final value, respectively and have been presented in Table 1. The fabricated devices display an ultrafast response upon exposure to humidity at room temperature. It is observed that the recovery time is much smaller than the response time. The time constants of the devices are limited by strong adsorption/desorption of the analyte gas molecules. The thermodynamic adsorption of water molecules on SnS_2_ films may not be much favorable at room temperature, therefore leading to a larger response time. Moreover, limited by our experimental setup, the introduction of humidity into the test chamber is very gradual. Therefore, the saturation of humidity (99% RH) takes a considerable time, which is reflected by the response times of our devices. On the other hand, during the recovery time, water molecules might be easily getting desorbed due to lower adsorption and the external flushing by dry nitrogen gas, resulting in a very fast recovery time^18^.

**Table 1:** Response and recovery times of the three devices at different biasing conditions (2–99% RH).

Sample/bias	0 V		1.0 V		2.0 V	
	t_r_ (s)	t_f_ (s)	t_r_ (s)	t_f_ (s)	t_r_ (s)	t_f_ (s)
Sample A	23.2	0.15	32.1	0.16	38.4	0.16
Sample B	10.4	0.72	13.2	0.87	20.9	0.88
Sample C	22	0.60	23.9	0.64	28.7	0.62

**Figure 5:**
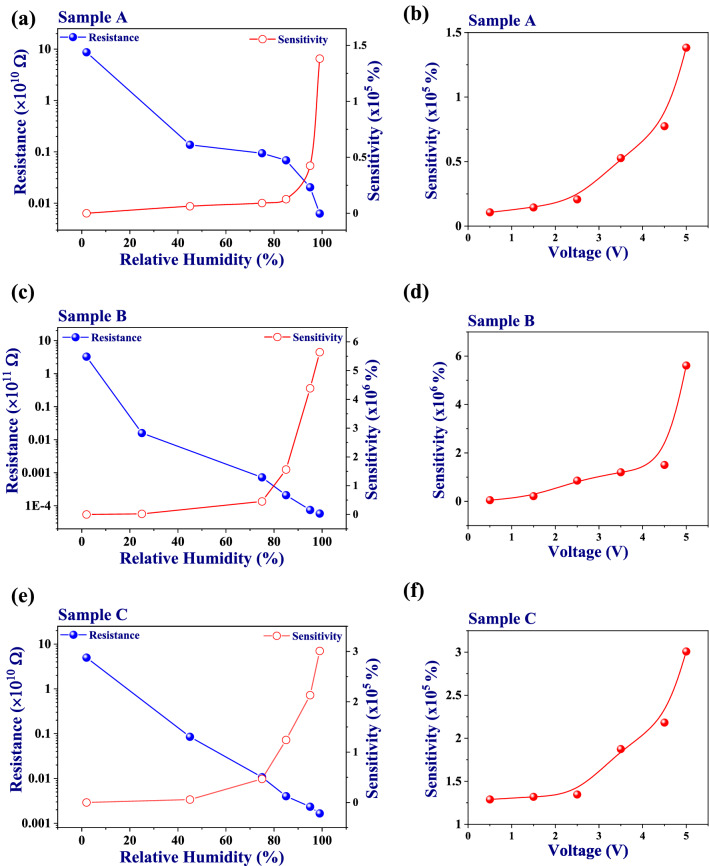
Resistance and sensitivity of (**a**) sample A, (**b**) sample B and (**c**) sample C as functions of RH at an applied bias of 5 V. Sensitivity as a function of applied bias for (**d**) sample A, (**e**) sample B and (**f**) sample C.

From the *I–V* measurements, the resistance values of the films have been calculated at 5 V. Figure 5a–c shows the plots of typical resistance values of the devices as a function of the relative humidity values. From these plots, it can be observed that the resistance decreased with increasing humidity. The sensitivity of the devices at different humidity levels have also been calculated using these resistance values. The sensitivity percentage can be calculated using the following expression^18^:

1 $$S\;(\% ) = \frac{{(R_{2} - R_{\Delta RH} )}}{{R_{\Delta RH} }} \times 100$$

where, R_2_ is the resistance of the sensor in 2% RH and R_ΔRH_ is the resistance of the device at the higher relative humidity level. The observed maximum sensitivities are ~ 1.38 × 10^5^%, ~ 5.64 × 10^6^% and 3.01 × 10^5^% for sample A, sample B, and sample C, respectively at 99% RH and 5 V applied bias (Fig. 5a–c). It is observed that sample B shows the highest sensitivity among the three samples. Sensing characteristics of a thin film sensor depends upon several factors such as its surface-to-volume ratio (SVR), surface roughness, porosity, initial resistance of the film, and so forth^31,32^. Increase in factors like SVR, roughness and porosity lead to increase in the number of adsorption sites. As the thickness of the film decreases, the SVR of the film increases. Therefore, for a thinner semiconducting film, the number of sites available for the adsorption of the analyte is expected to be more, which results in higher sensitivity of the sensor. However, in the present study, sample B shows the highest sensitivity. The possible reason could be the higher surface roughness of sample B. Higher surface roughness increases the SVR and more exposed sites for humidity adsorption, therefore resulting in increased sensitivity. Interestingly, the root mean square (RMS) roughness of the samples (Figure S2 of the Supplementary Information) follow the order sample A (101 nm) > sample B (78.8 nm) > sample C (21 nm), indicating thereby that the surface roughness of sample B is much higher than the surface roughness of sample C. It can also be seen from Fig. 5a–c that the initial resistance value of sample B is the highest among the three sensors, and therefore, the combined effect of all the factors mentioned above results in the highest sensitivity for sample B. An important figure of merit of any sensor is its limit of detection (LoD), which reveals the minimum concentration of analyte gas that can be detected significantly. The LoD has been estimated for sample B, and it comes out to be ~ 4.9% RH. The details have been given in Figure S3 of the Supplementary Information.

Furthermore, we can also explain the difference in the response/recovery times of the sensors with different film thicknesses. The response is faster in case of sample B, as it has the highest number of exposed sites so that greater number of water molecules can be detected in a shorter span of time. However, the weak desorption of water molecules (chemisorbed or physisorbed on the semiconductor surface) leads to the slower recovery as compared to the other samples. Figure 5d–f shows the variation in the sensitivity of the devices with the applied voltage. It can be seen that with increase in the bias voltage, the sensitivity increases for all the devices due to establishing the high current at the electrodes. The charge carriers contributing to the current are effectively collected by the electrodes, thus leading to a high current and hence, higher sensitivity.

**Figure 6:**
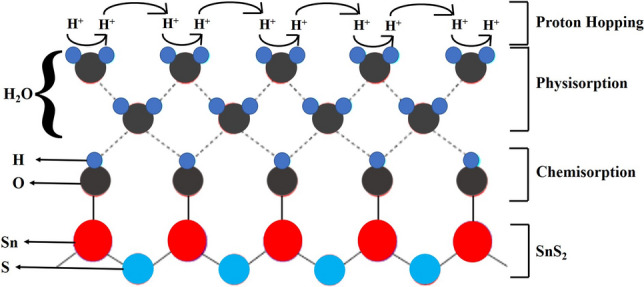
Humidity sensing mechanism showing proton hopping. Schematic of Grotthusss chain reaction for protonic conduction on the surface of SnS_2_ (inspired from Ref.^33^).

The sensing mechanism can be explained as follows. The conductivity of an n-type semiconducting device is due to presence of electrons. Under lower humid conditions, electronic conductivity prevails as protons (H^+^ ions) cannot move freely in the immobile chemisorbed and the first physisorbed layers of water molecules^3^. With increase in humidity levels (> 70% RH), the H^+^ ions can move freely in higher-level layers of physisorbed water molecules according to the Grotthuss reaction (Fig. 6), resulting in the dominant protonic conduction. With further increase in humidity levels (> 90% RH), the formation of physisorbed water molecule layers increase in number that results in a huge number of freely moving H^+^ ions, which leads to an increase in the protonic conduction. Therefore, the sensor’s response enhances with increase in humidity. In addition to this, the previously adsorbed oxygen species also play a crucial role in defining the initial and final conductivity of the device. Thus, the final conductivity is the result of the competitive mechanisms of adsorption and desorption of the water molecules as well as the oxygen species^33^.

**Figure 7:**
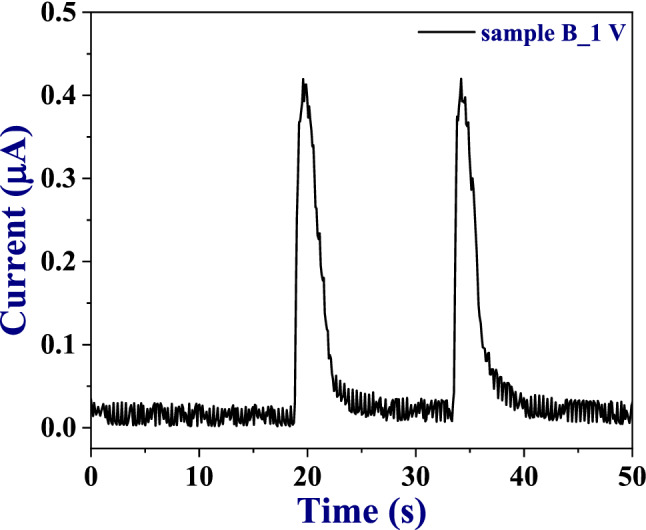
Breath test on sample B at 1 V applied bias.

One of the most important aspects of any sensor is its performance in ambient conditions, which reflects its usability for practical applications. To test the performance of our devices in ambience, we have carried out breath test on two of the devices (sample A and sample B) by gently exhaling on the sample, kept ~ 4.0*–*4.5 cm away from the mouth. As representative results, the breath detection of sample B is shown in Fig. 7. The response time is ~ 1 s whereas the recovery time is estimated to be ~ 2*–*3 s. It has been observed that the response/recovery time during the breath test decreased/increased as compared with values in Table 1. The decrease in the response time is because humidity exposure is instantaneous in breath test whereas in our experimental setup humidity increases gradually, whereas the recovery time is affected by the absence of external N_2_ flushing. Such fast and efficient sensors have a huge potential to be used for realization of practical devices, such as in the field of biomedical applications.

The performance of these SnS_2_ nanofilms devices is extremely well compared to other 2D materials-based humidity sensors reported in literature^1,18,34–41^. A comparison of the sensing characteristics of our devices with other reported devices has been presented in Table 2. The fabricated SnS_2_-based ultrafast and self-powered humidity sensors are robust and low cost that might open routes for novel applications such as in breath monitoring. The self-powered behavior of our sensors can be extremely useful in systems which involve an array of such sensors, and therefore, the operational cost and the heavy circuitry becomes a big concern in the current scenario of energy crisis. Our findings may also enable nano-porous thin film-based sensors to exploit the unique advantages unveiled from the ultrathin nature of the constituent sheets and the facile synthesis processes, suitable for applications in fields such as consumer’s electronics, environmental and health monitoring.

**Table 2:** Comparison of sensing properties of various state-of-the-art humidity sensors reported in literature with the present work.

Materials	Bias (V)	Sensitivity (%)	Response time (s)	Recovery time (s)	References
SnS_2_: Sample A	0	–	23.20	0.15	This work
	1	1.19 × 10^4^	32.10	0.16	
Sample B	0	–	10.40	0.72	
	1	1.54 × 10^5^	13.20	0.87	
Sample C	0	–	22	0.60	
	1	1.31 × 10^5^	23.90	0.64	
Black Phosphorus	–	~ 99	255	10	^36^
MoS_2_	–	~ 30	140	80	^35^
Graphene Oxide	1	37,800	10.5	41	^34^
MoS_2_	–	3	9	17	^38^
VS_2_	1	> 300	30–40	12–50	^37^
SnS_2_	3	11,300	85	6	^18^
WS_2_	5	–	140	30	^1^
TiO_2_	0	–	4.5	2.8	^39^
ZnO	0	43.4	70	–	^40^
Ga doped ZnO	0	358	< 5	–	^41^

## Conclusions

We have successfully fabricated 2D SnS_2_ thin films on soda lime glass substrates by a two-step synthesis process*—*dc sputtering of Sn films followed by their sulfurization. XRD and Raman spectra confirmed the hexagonal crystal structure and SEM–EDS micrographs confirmed homogeneity and uniformity of SnS_2_ films over a large area. Humidity sensing measurements of SnS_2_ thin film-based devices of different thicknesses exhibit a highly responsive detection at 0 V for all the devices, showing self-powered nature of the devices. Temporal response was done at different bias voltages (0*–*2 V) and it is observed that the devices show high electrical stability and repeatability in ambient conditions. From the transient response curves, ultrafast response and recovery times of ~ 10 s and ~ 0.7 s, respectively, have been estimated for the self-powered detection of the sensor with optimum film thickness. Furthermore, a maximum sensitivity of ~ 5.64 × 10^6^% in 99% RH at an applied bias of 5 V can be achieved. These results could pave a way for designing self-powered and ultrafast humidity sensors with simple architectures through easy approaches.

## Methods

### Thin film fabrication

SLG substrates were first sonicated for 15 min in iso-propyl alcohol followed by sonication in deionized water for 10 min. The substrates were then purged with nitrogen gas to remove any residuals before being loaded into the sputtering chamber (Excel Instruments). The thin films were prepared by a two-step process which involved: (i) deposition of Sn thin films on SLG by dc magnetron sputtering of Sn target (3 inch in diameter) at 45 W—15 min (sample A), 5 min (sample B), 1 min (sample C) and (ii) sulfurization (sulfur powder by Sigma Aldrich, 99.98%) at 450 °C for 1 h in a tube furnace with continuous Ar gas flow. The sulfurization process has been explained in our previous report^42^. All the deposition parameters were optimized to obtain best conditions for continuous thin film and pure phase.

### Thin film characterization

The crystal structure of the prepared films was examined by X-ray diffraction (XRD)—X’pert-PRO PANalytical with Cu Kα radiations (1.5418 Å). The surface morphology and compositional analysis of the films were determined using a scanning electron microscope (SEM)—Ultra55 FE-SEM Karl Zeiss—EDS. Thickness of the films were measured by cross-sectional SEM. Atomic force microscopy (AFM) was carried out using A.P.E. Research A100-AFM. Raman analysis was done using LabRAM HR equipped with a 532 nm laser.

### Device fabrication and humidity sensing

For humidity sensing measurements, 120 nm thick Cr/Au (10 nm/110 nm) contacts were deposited on the SnS_2_ films to make metal–semiconductor–metal type contacts. A very simple computer-controlled setup which consisted of a test chamber, a mass flow controller, a source for humid air, and a data acquisition system was employed for humidity sensing measurements. During measurements, humid air was obtained by passing nitrogen gas through water and the flow was controlled with a mass flow controller. The conductivity of the samples was then measured with a Keithley 2400 source measuring unit, in different RH conditions.

## Supplementary information

Supplementary Information.
