# Supplementary material for: Self-powered, ultrasensitive, room temperature humidity sensors using SnS2 nanofilms
Source: Sci Rep. 2020 Sep 3;10:14611. doi: 10.1038/s41598-020-71615-5 (PMC7473760; doi:10.1038/s41598-020-71615-5)
Supplement: Supplementary file 1 — Supplementary Information. [file 41598_2020_71615_MOESM1_ESM.docx]

**Supplementary Information**

# **Self-Powered, Ultrasensitive, Room Temperature Humidity Sensors using SnS_2_ Nanofilms**

*A. Rambabu^a†*^, Deependra Kumar Singh^b†^, Rohit Pant^b^, K. K. Nanda^b^, S. B. Krupanidhi^b*^*

^a^ Department of Basic Science and Humanities, GMR Institute of Technology, Rajam

Andhra Pradesh - 532127, India

^b^ Quantum Structures and Device laboratory, Materials Research Centre, Indian Institute of Science, Bangalore - 560012, India

**^†^** These authors contributed equally to this work.

* Corresponding authors: [sbk@iisc.ac.in](mailto:sbk@iisc.ac.in), [rambabuhcu@gmail.com](mailto:rambabuhcu@gmail.com)


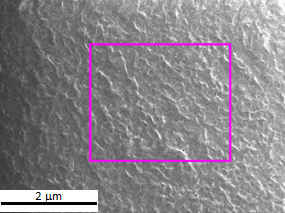

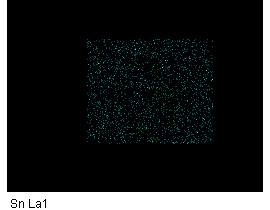

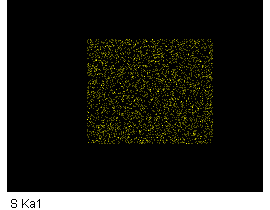

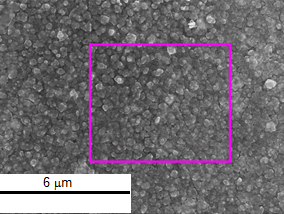

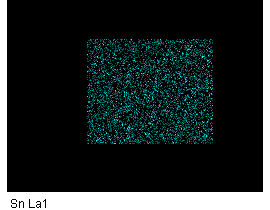

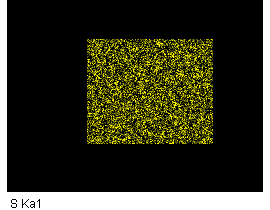

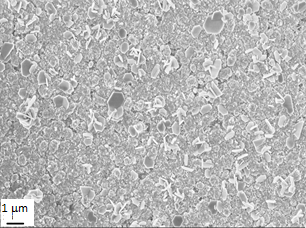

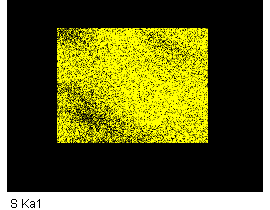

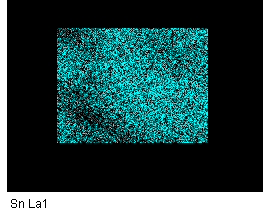


**(a)**

**(b)**

**(c)**

**(d)**

**(e)**

**(f)**

**(g)**

**(h)**

**(i)**

**Figure S1**. SEM-EDS micrographs of (a-c) sample A, (d-f) sample B, and (g-i) sample C, showing the uniform and homogenous distribution of Sn and S.


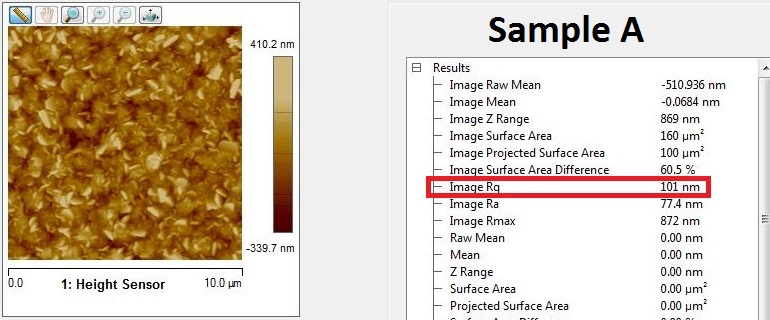

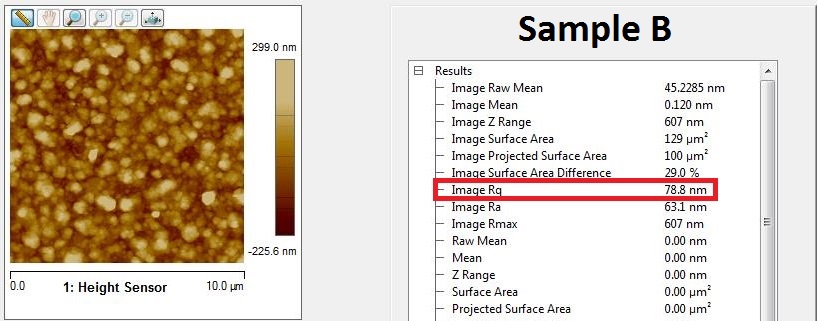

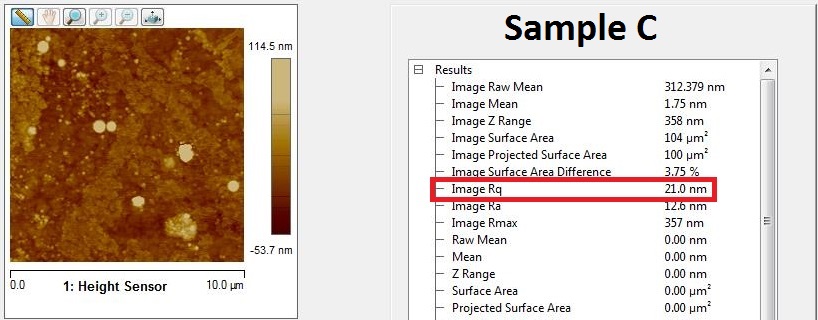


**Figure S2**. AFM scans of sample A, sample B, and sample C (indicated in the figure), showing the RMS roughness values.

**Figure S3**. Linear fitting of the lower humidity region of the sensitivity vs %RH curve for sample B.

Figure S3 shows the linear fitting of the lower humidity region of the sensitivity vs %RH curve for sample B. IUPAC defines the limit of detection (LoD) of a sensor as the minimum concentration of the analyte gas that can be detected significantly and with reasonable certainty for a given analytical procedure.^1,2^

$$LoD=\frac{k⨯\sigma_{D}}{m}$$

where, $k$ is a constant whose value has been taken as 3, $\sigma_{D}$ is the standard deviation, and $m$ is the slope of the calibration line. The LoD for sample B comes out to be ~4.9 %RH.

1. Honeycutt, W. T., Ley, M. T. & Materer, N. F. Precision and limits of detection for selected commercially available, low-cost carbon dioxide and methane gas sensors. *Sensors* **19**, 3157 (2019).
2. Long, G. L. & Winefordner, J. D. Limit of Detection: A Closer Look at the IUPAC Definition. *Anal. Chem.* **55**, 712A–724A (1983).
